# A highly sensitive wearable pressure sensor capsule based on PVA/Mxene composite gel

**DOI:** 10.1007/s13205-022-03221-3

**Published:** 2022-07-13

**Authors:** Nithusha Kallingal, Muni Raj Maurya, M. S. Sajna, Huseyin Cagatay Yalcin, Hassen M. Ouakad, Issam Bahadur, Somaya Al-Maadeed, Kishor Kumar Sadasivuni

**Affiliations:** 1 Center for Advanced Materials, Qatar University, P.O. Box 2713, Doha, Qatar; 2 Department of Mechanical and Industrial Engineering, Qatar University, P.O. Box 2713, Doha, Qatar; 3 Biomedical Research Center, Qatar University, P.O. Box 2713, Doha, Qatar; 4 Mechanical and Industrial Engineering Department, College of Engineering, Sultan Qaboos University, Al-Khoudh, P.O.-Box 33, 123 Muscat, Oman; 5 Department of Computer Engineering, Qatar University, P.O. Box 2713, Doha, Qatar

**Keywords:** PVA/Mxene, Sensor capsule, Wearable, Pulse detection

## Abstract

Wearable sensors have drawn considerable interest in the recent research world. However, simultaneously realizing high sensitivity and wide detection limits under changing surrounding environment conditions remains challenging. In the present study, we report a wearable piezoresistive pressure sensor capsule that can detect pulse rate and human motion. The capsule includes a flexible silicon cover and is filled with different PVA/MXene (PVA-Mx) composites by varying the weight percentage of MXene in the polymer matrix. Different characterizations such as XRD, FTIR and TEM results confirm that the PVA-Mx silicon capsule was successfully fabricated. The PVA-Mx gel-based sensor capsule remarkably endows a low detection limit of 2 kPa, exhibited high sensitivity of 0.45 kPa^−1^ in the ranges of 2–10 kPa, and displayed a response time of ~ 500 ms, as well as good mechanical stability and non-attenuating durability over 500 cycles. The piezoresistive sensor capsule sensor apprehended great stability towards changes in humidity and temperature. These findings substantiate that the PVA/MXene sensor capsule is potentially suitable for wearable electronics and smart clothing.

## Introduction

The pulse wave is a physiological phenomenon generated in the heart. The arterial blood vessels circulate this pulse wave to the limps and different parts of our body. The pulse wave varies from one person to another, which indicates the fitness of our heart and blood vessels. From pulse waves, we can monitor the oxygen level in the blood, blood pressure, and heart rate. Pulse rate sensors are commonly used in medical gear, and they can be attached to any place, where the pulse rate is strong such as the wrist, on the elbow, neck sides, and on the foot top (Ma et al. 2017). Pulse rate sensors detect the heart pulse and convert it into electrical signals. Previously, sensors were made up of conductive materials like metals and semiconductors (Lewis and Ahn 2015). However, flexible sensors such as silicone-based are non-conducting in nature and have good biocompatibility as well as excellent flexibility, which also shows excellent response compared to conducting materials (Pang et al. 2012).

Polymers modification with 2D conductive material fillers is a recent trend in wearable sensors, enhancing the sensor's physical, chemical, and structural stability in an unpredictable environment (Zhao et al. 2021). In addition, 2D layered materials such as graphene oxide, MoS_2_, and carbon nanotubes, have been introduced into polymers (Qadir et al. 2021; Li et al. 2020). Recently, a new kind of 2D transition metal carbide, nitride and carbonitrides materials, also known as MXene (Mx), has been widely studied for sensor applications (Pei et al., 2021). MXene (M_*n*+1_AX_*n*_) is a two-dimensional material with exceptional physical, chemical, and electrical properties. It belongs to the family of 2D transition metal carbides, carbonitride. In M_*n*+1_AX_*n*_, the M is represented by the early transition metal (Sc, Ti, V, Cr, Mo, etc.), A denotes the elements belonging to the groups 13 or 14 (Al, Si, Sn, In, etc.), X can be either Carbon or Nitrogen, and *n* will differ from 1 to 3, the subsequent single MXene sheets contain 3, 5, or 7 atomic layers for M2X, M3X2, and M4X3, respectively. Due to its excellent mechanical stimuli-responsive property with hydrophilicity and conductivity, MXene has been used in wearable–flexible–piezoresistive–pressure and humidity sensors (Wang et al. 2019, 2021; Jiang et al. 2018). The report reveals that adding polyvinyl alcohol (PVA) to MXene can decrease the diffusion coefficient of MXene and boost the chemical stability of the composite (Zhao et al. 2021). It also stabilizes the performance of the composite over half a year. Moreover, PVA is an inexpensive polymer material used with carbon-based fillers because of its nontoxic property, durability, and quick processability (Nan and DeVallance 2017). Furthermore, the conductivity of MXene and internal resistance are highly dependent on its compressible sheets-laminated structure. In the 2D MXene film-based piezoresistive sensor, the deformation limit of MXene is quickly reached due to the limited space of 2D plane structure. As a result, the sensor’s stability and response get restricted under force. In addition, most of the reported studies have focused on the sensitivity or limit of external stimulation. Moreover, the sensor response under changing surrounding conditions such as humidity and temperature was not analyzed.

Herein, we report a novel capsule confined PVA-MXene (PVA-Mx) gel-based multi-functional flexible piezoresistive pressure sensor. The silicone capsule has high hydrophobicity, good thermal stability, corrosion resistance and high flexibility that holds the gel inside the capsule and offers a good composite flow, providing better aesthetics. Ascribing to the capsule-confined effect, PVA/MXenes gel endows larger deforming space and more sensitive micromotion ability. During external stimulation, both the distance between neighboring interlayers in MXenes and different MXenes will decrease with respect to degrees of applied external force, resulting in a corresponding change in the resistance. Due to this elastically confined nature, the pressure sensor capsule exhibits high sensitivity (0.45 kPa^−1^), relatively short response time (~ 500 ms), and great reproducibility over 500 cycles. Moreover, the reported piezoresistive sensor could achieve multi-functional sensing, such as pressure and motions by one device. The simple preparation method and capsule-based design make the current sensor applicable for large-scale production. Based on the above sensing performance, the PVA/MXene gel-based sensor capsule has potential application in medical monitoring, human activity recognition and other sensitive information collection.

## Materials and methods

### Materials

MXene (Ti_3_C_2_T_x_) was purchased from Sigma Aldrich. Polyvinyl Alcohol is purchased from Gulf Energy Technology & Projects W.L.L.(Qatar), manufactured by PolyChem, Hongkong, China. Silicone resin was purchased from Smooth-On, Texas, U.S.A.

### Fabrication of PVA/MXene—silicone capsule sensor

**Fig. 1:**
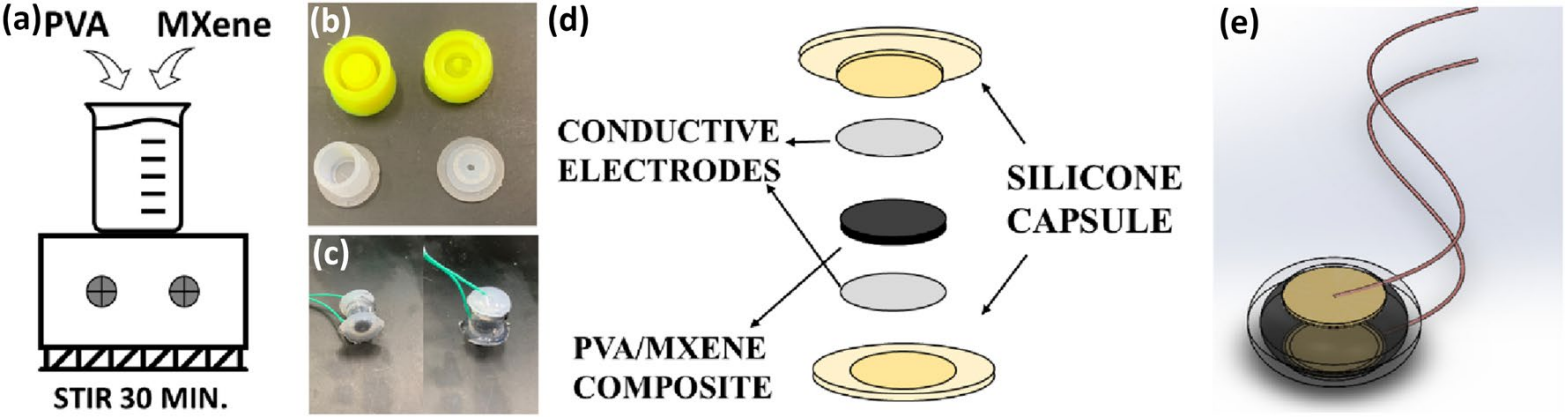
**a** Preparation of PVA-Mx composite gel. **b** 3D-printed mold (top) and casted silicone capsule (bottom). **c** Photograph of fabricated PVA-Mx sensor capsule. **d** Schematic representing the configuration of PVA-Mx sensor capsule. **e** Schematic illustration of the sensor

The PVA-Mx composite with MXene loading of 0.5 wt%, 1 wt%, 1.5 wt%, 2 wt% and 2.5 wt% were prepared by varying the concentration of MXene in PVA solution. Figure 1 shows the schematic illustration of the steps involved in sensor fabrication. The composite is stirred homogeneously for 30 min at room temperature using a magnetic stirrer for uniform dispersion. The silicon capsule is prepared by mixing a 1:1 silicone part A and part B (crosslinker). After that, the silicone mixture was transferred to the 3D-printed mold. It was kept without disturbance for 40 min to attain the crosslinking. After curing, the silicone capsule is removed from the mold. Two electrodes are connected in the two ends of the capsule, which are prepared using a strip of aluminum foil and a copper wire. The silicone capsule was filled with the prepared solutions of PVA/MXene composite. The schematic of the fabricated PVA-Mx sensor capsule is shown in Fig. 1d, e.

### Characterization

The PVA/MXene composites were cast into films, and the surface morphology and structure of prepared composite films (0.5 wt%, 1wt%, 1.5 wt%, 2 wt% and 2.5 wt%) and PVA were evaluated using TEM (FEITECNAI G2 TEM, TF20). XRD of all the prepared PVA-Mx films, PVA and MXene alone were performed on the model Rigaku, Miniflex2 Desktop, Tokyo, Japan, with Cu Kα radiations. The composition of PVA-Mx composites and PVA was assessed by Fourier Transform Infrared Spectroscopy (FTIR). The attenuated total internal reflection method was adopted for this test.

### Sensor testing

To evaluate the response of the sensor under loading and unloading of different pressure, a setup consisting of a Keysight-Function arbitrary waveform generator (20 MHz), vibrating plate and source meter unit (Keithley 2612A) was used. Under varying forces, the sensor response was evaluated by applying an AC sine wave signal with a peak amplitude of 100 mV at different frequencies (600 mHz, 800 mHz, 1 Hz, 1.5 Hz, and 2 Hz). The data acquisition was accomplished through LabView software, and relative changes in resistance vs. time graph were plotted to monitor the sensor response. The relative change in resistance (*A*_R_) of composites was calculated using the following equation (Eq. 1):

1 $$A_{{\text{R}}} \left( \% \right) \, = \, \Delta R/R_{0} \times \, 100$$

$$\Delta R \, = \, R_{0} - R$$

Here, the resistance of the composites under no pressure and with applied pressure is represented as *R*_0_ and *R*, respectively. The relative change in resistance in percentage (A_R_ (%)) was quantitatively evaluated in each sample using Eq. 1.

## Results and discussion

**Fig. 2:**
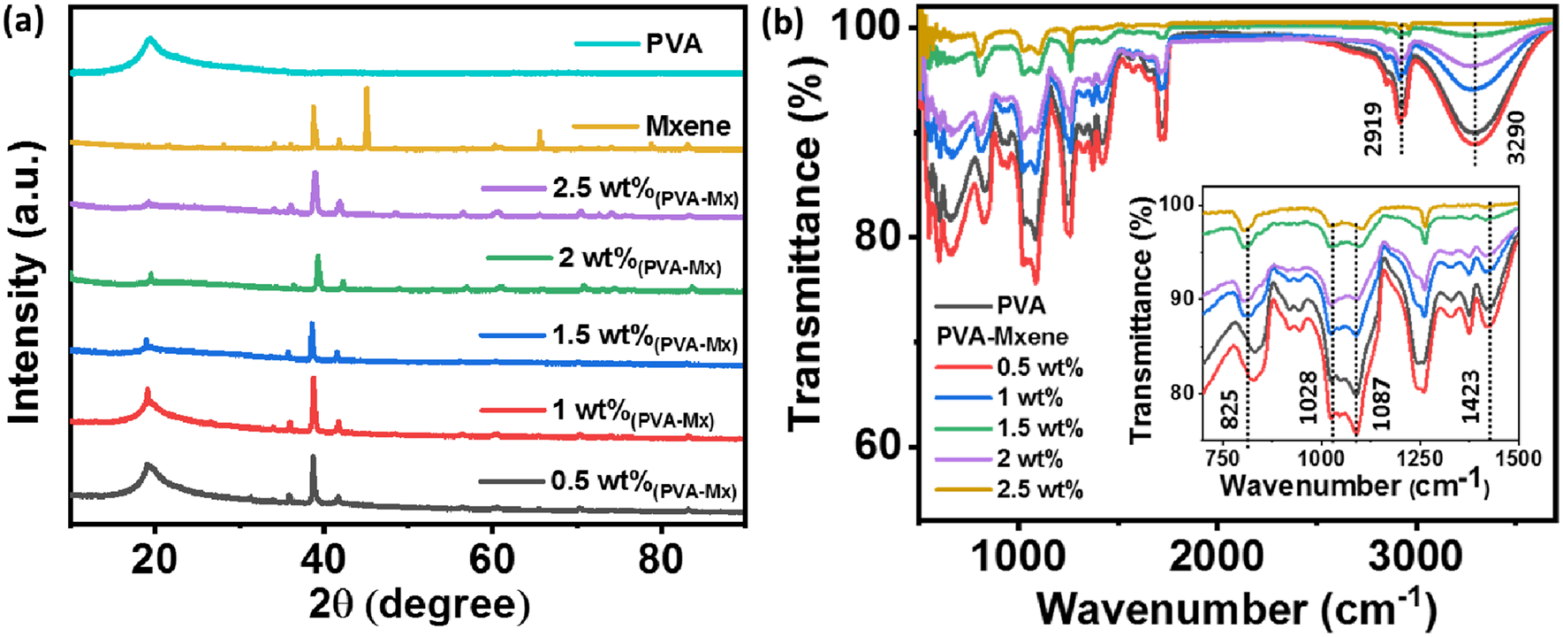
**a** XRD spectra of samples with different weight percentage (0.5–2.5 wt%) loading of MXene in PVA. **b** FTIR plot of the samples with different weight percentages (0.5–2.5 wt%) loading of MXene in PVA

The XRD spectra of the PVA, MXene and PVA-Mx composites are shown in Fig. 2a. MXene spectra have diffraction planes of (100), (104) and (1011), which is equivalent to the peaks at 2*θ* values of 34.0°, 39.0° and 65.6°, respectively, of the MAX Ti_3_AlC_2_ (JCPDS 52-0875). These values are very similar to the earlier literature reports (Tang et al. 2016). XRD pattern of PVA indicates unimodal with a peak at 2*θ* value of ~ 19°, which implies that it is a high molecular-weight polymer. With the increase in the loading percentage of MXene in PVA, the PVA peak at 2θ of ~ 19° tends to disappear. However, the distinctive peaks of MXene are evident in all PVA-Mx composites (Fig. 2a). Thus, it suggests that the MXene is very effectively blended in the PVA solution (Pan et al. 2020). Furthermore, the structural characterizations of different PVA-Mx composites and PVA were analyzed by FTIR spectroscopy in the range of 500–4000 cm^−1^, as shown in Fig. 2b. The peaks at 3290 cm^−1^, 2919 cm^−1^, 1423 cm^−1^, 1087 cm^−1^, 1028 cm^−1^, and 827 cm^−1^ are common for both PVA and PVA-Mx composites. In the absorption peak of PVA, an OH^−^ stretching of hydroxyl groups is visible at 3290 cm^−1^ because of the inter–intra molecular hydrogen bonding transpired in the PVA strings (Kharazmi et al. 2015). The symmetric C–H bonding and CH_2_ bending can be evident at 2919 cm^−1^ and 1423 cm^−1^, respectively. In addition, an absorption peak of PVA C–O stretching and O–H bending can be found at 1087 cm^−1^ and 1028 cm^−1^. The peak corresponding to the rocking (PVA) can be seen at 860 cm^−1^. The MXene-influenced peaks are not reflected in the graph, and no extra peaks are found in the spectrum with different weight percentage loading of MXene in PVA. The identical curves confirm that physical interaction happens among the components and no other interactions are observed in between PVA and MXene.

**Fig. 3:**
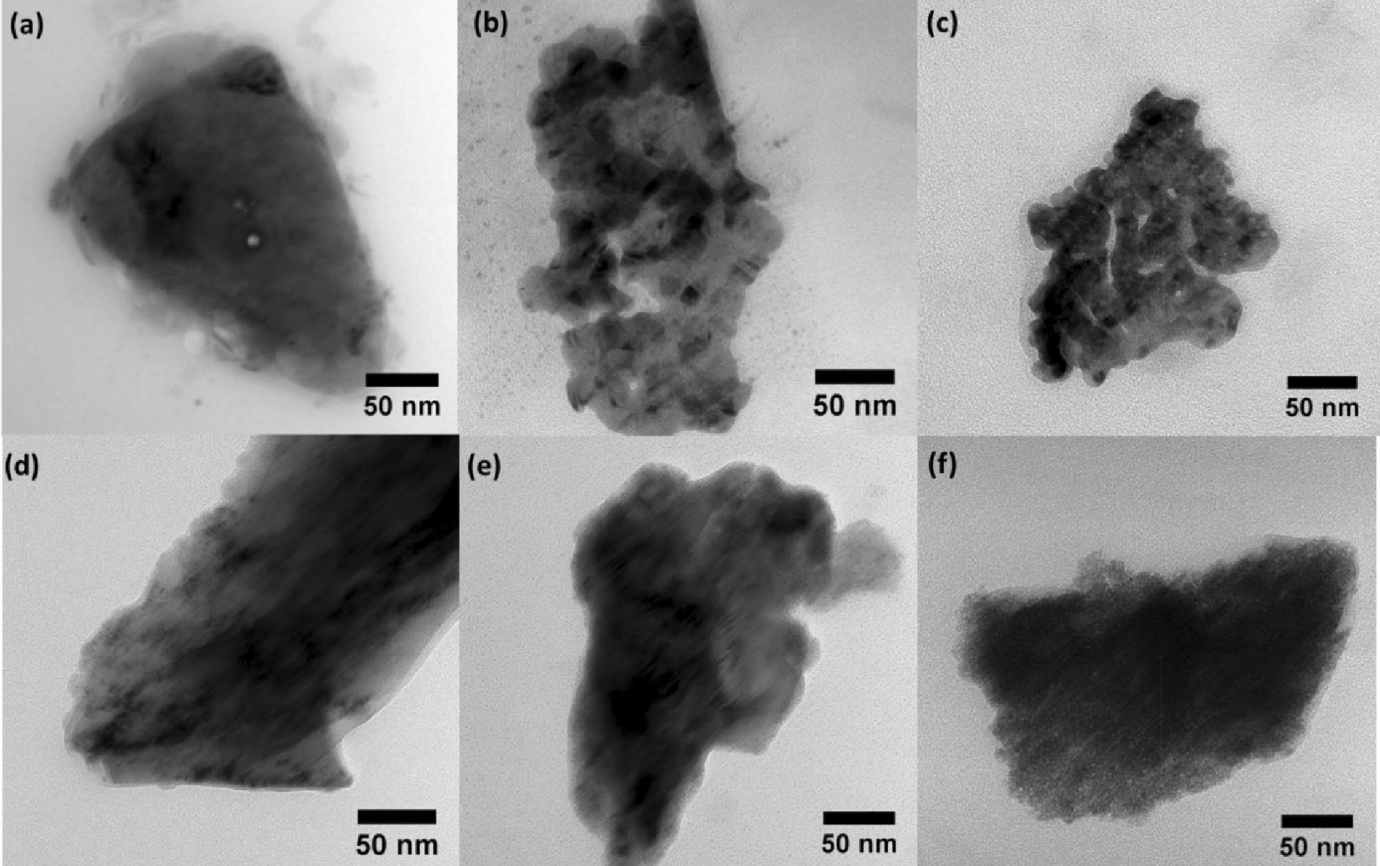
TEM image of the samples. **a** PVA. **b** 0.5 wt% PVA-Mx composite. **c** 1 wt% PVA-Mx composite. **d** 1.5 wt% PVA-Mx composite. **e** 2 wt% PVA-Mx composite. **f** 2.5 wt% PVA-Mx composite

The surface morphology of PVA and PVA-Mx composites in various concentrations were studied using TEM analysis, as shown in Fig. 3. The TEM image of bare PVA is presented in Fig. 3a. For samples loaded with 0.5 wt% and 1 wt% MXene in PVA, dark spots were observed in the TEM images (Fig. 3b, c). These dark spots represent MXene in the PVA-Mx composite and are randomly distributed in the form of clusters. This confirms that low wt% loading of MXene in PVA leads to accumulation of filler in the polymer matrix. However, with an increase in the wt% of MXene from 1.5 to 2.5 wt%, uniformity of the dark region in the PVA matrix increases, as shown in Fig. 3c–e, respectively. The 2.5 wt% MXene loaded sample exhibited a highly uniform distribution of the filler. Remarkably, MXene overlapped on the surface of PVA and decked out the PVA very well. Hence, this distinctive morphology of the 2.5 wt% PVA-Mx sample may increase the conductivity of the composite and can facilitate high-pressure sensitivity.

**Fig. 4:**
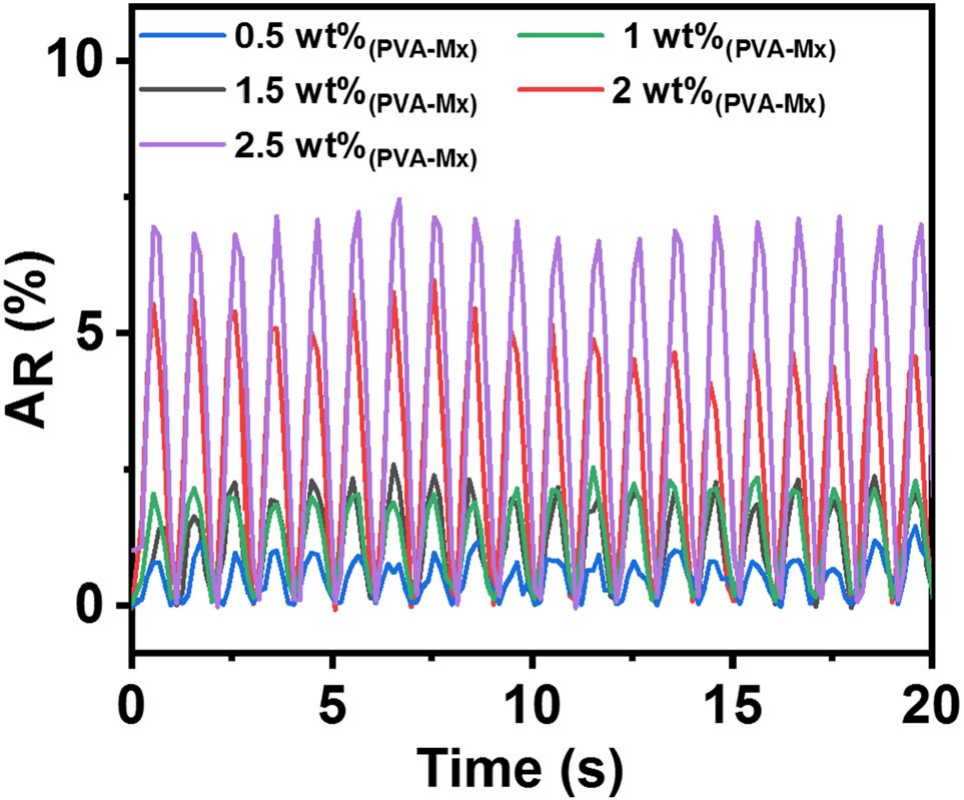
Response of piezoresistive sensor capsules fabricated by varying the MXene weight percent (0.5–2.5 wt%) in PVA. The response was measured under loading and unloading of 10 kPa pressure at a frequency of 1 Hz and a constant bias of 2 V

Response of the piezoresistive sensor capsules fabricated by varying the weight percent of MXene in PVA was examined under loading and unloading of 10 kPa at a frequency of 1 Hz and constant DC bias of 2 V, as shown in Fig. 4. When the pressure of 10 kPa is applied, the gap between MXene flakes dispersed in PVA becomes smaller, and the resistance of the PVA-Mx gel decreases. Subsequently, with the release of applied pressure, the gap between MXene flakes increases, resulting in an increase of resistance. Compared to other sensor capsules, the sensor fabricated with 2.5 wt% MXene exhibited the highest response and an increase in response was observed with the increase in MXene wt% in PVA. Under low loading of MXene in PVA, i.e., 0.5–1.5 wt%, the gap between adjacent flakes is more, leading to poor contact between flakes under pressure, and sensors display low response. Furthermore, with the increase in MXene loading in PVA, i.e., 2 wt% and 2.5 wt%, the gap between adjacent flakes decreases and a significant increase in response is noticed, as shown in Fig. 4. However, with MXene loading above 2.5 wt%, sensors displayed significantly low resistance when there is no pressure, indicating the formation of continuous conducting channels and negligible change in resistance was noticed under loading and unloading of pressure. The above investigation shows that the sensor capsule having PVA loaded with 2.5 wt% MXene is highly preferable as a piezoresistive sensor.

**Fig. 5:**
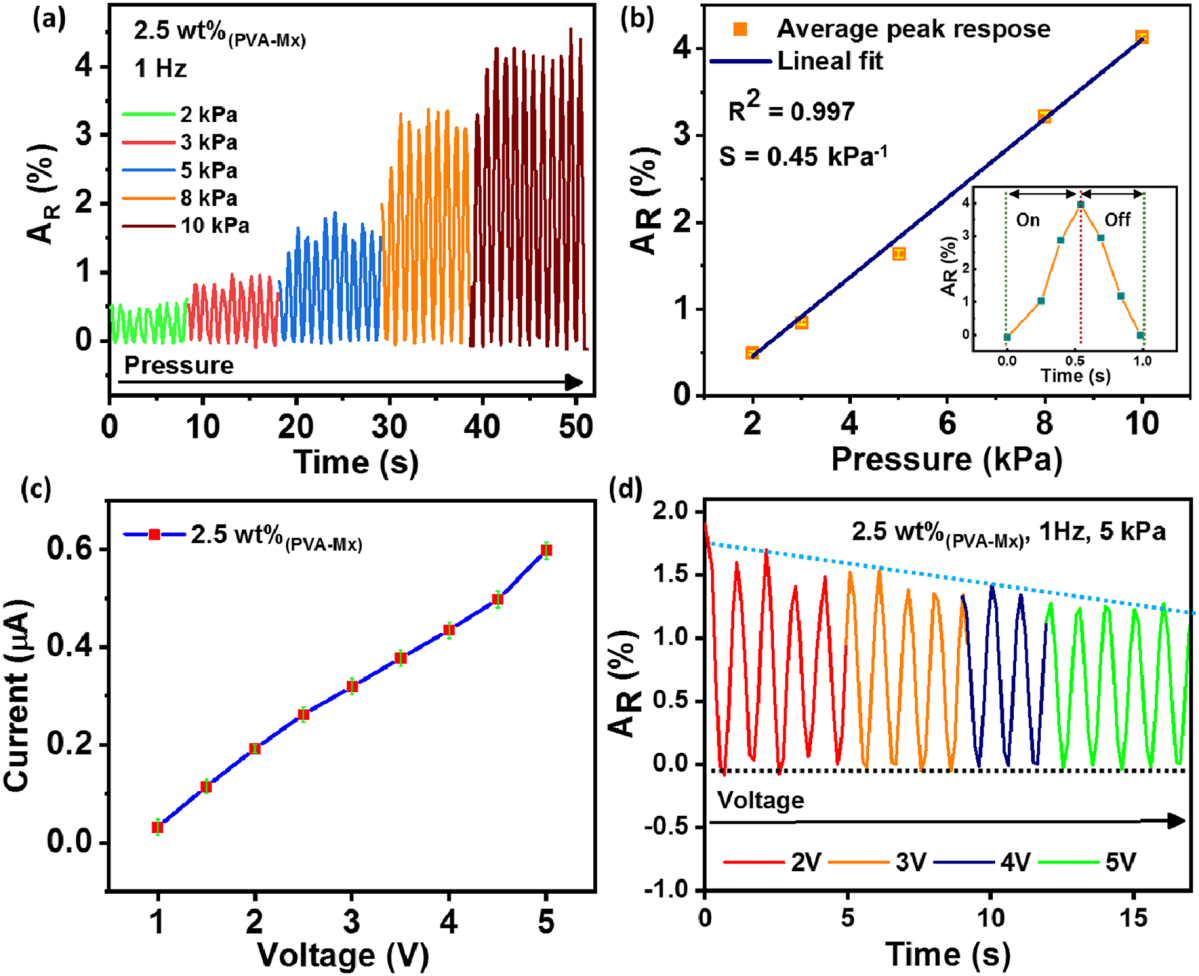
Response of the PVA-Mx composite-based sensor capsule. **a** *A*_R_ vs. time graph of 2.5 wt% PVA-Mx sensors under different pressures (2 kPa, 3 kPa, 5 kPa, 8 kPa, 10 kPa) at a constant frequency of 1 Hz and DC bias of 2 V. **b** Relative change of resistance with pressure. The inset figure shows an amplifying *A*_R_ vs. time curve part exhibiting a fast response time of ~ 500 ms. **c** Linear relation of the current vs. voltage *I*–*V* plot under no pressure indicated the Ohmic contacts between 2.5 wt% PVA-Mx composite and capsule electrodes. **d** *A*_R_ vs. time plot of 2.5 wt% PVA-Mx sensors under 5 kPa at a constant frequency of 1 Hz and DC bias of 2 V

The response properties of the 2.5 wt% PVA-Mx composite-based sensor capsule were analyzed in the pressure range of 2–10 kPa. Figure 5a shows the response of the piezoresistive pressure sensor under different pressure loadings at the frequency of 1 Hz and a DC bias of 2 V. With the serial increase in the pressure, a gradual increase in the relative resistance was observed, suggesting that the sensor conductivity increased, and the total resistance decreased accordingly. Thus, the sensors can easily distinguish the strength of the applied pressure by judging the magnitude of change in relative resistance. The sensor capsule displayed a stable and noise-free response, indicating the high stability and repeatability of the sensor. The sensitivity of the piezoresistive sensor is an important parameter for evaluating device performance and is evaluated by Eq. 2 (Li et al. 2019):

2 $$S \, = \delta \left( {\Delta R/R_{0} } \right)/\delta P,$$

where *δ*(Δ*R*/*R*_0_) and δ*P* represent the relative change in resistance and pressure variation, respectively. The linearity of the curve (Fig. 5b) shows that the sensor capsule apprehends an excellent linear response under corresponding pressure ranges. The estimated sensitivity was 0.45 kPa^−1^, making it capable of detecting sensitive human movements. Another important parameter of the sensor is the response time, and fast response without hysteresis can ensure a timely response under external pressure. The inset graph in Fig. 5b shows the enlarged response cycle of the sensor under loading and unloading of 10 kPa. The sensor exhibits response and recovery times of ~ 500 ms under 10 kPa, which are sufficient to meet the requirements of real-time applications. Electrical conductivity is an essential property of a sensor. The plot of current vs. voltage of sensor capsule under no pressure loading is shown in Fig. 5c. It is evident from Fig. 5c that the current is increasing with respect to the voltage in an almost linear relation. It suggests that 2.5 wt% PVA-Mx composite gel has Ohmic contact with the two electrodes (Gao et al. 2020). The linear relationship in Fig. 5c indicates that resistance will remain almost constant for a given pressure and applied voltage. Thus, the sensor response will be guided by the change in resistance of the PVA-Mx gel under loading and unloading of external stimulation. Thus, the relative resistance of the sensor can be linearly increased with the increase of external force, as depicted in Fig. 5b. To further confirm the Ohmic contact of composite with the electrodes, the sensor response with an increase in voltage was examined under loading and unloading of 5 kPa pressure, as shown in Fig. 5d. The sensor displayed a linear decrease in the relative change of resistance with an increase in the voltage, establishing that the sensor could sustain the Ohmic contact with electrodes under pressure loading and unloading.

**Fig. 6:**
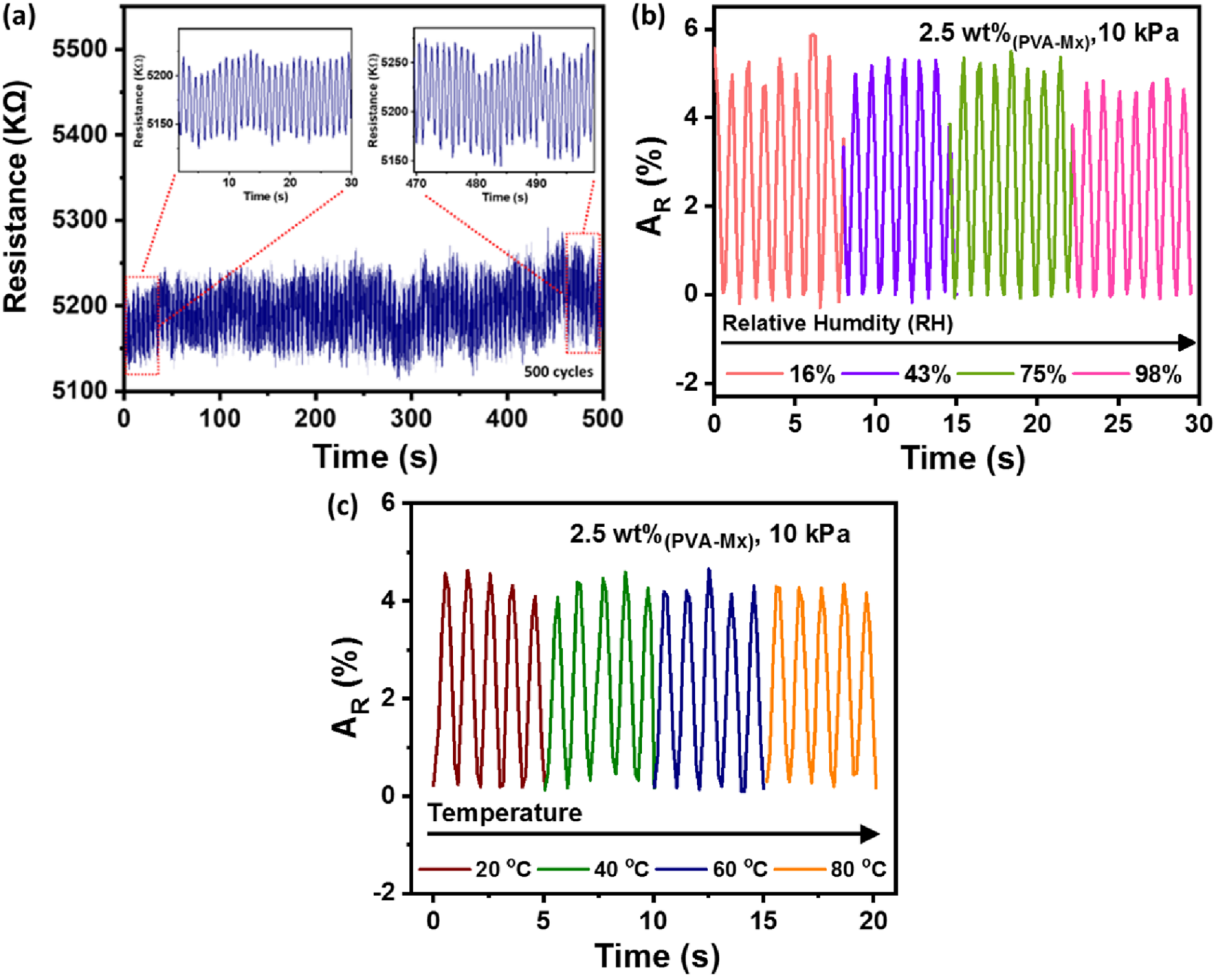
**a** Mechanical durability and repeatability response of 2.5 wt% PVA-Mx sensor capsule under loading and unloading of ~ 10 kPa over 500 cycles. **b** *A*_R_ vs. time graph of samples 2.5 wt.% PVA-Mx sensor at different relative humidity (16%,43%, 75%, and 98% RH), under 10 kPa pressure at a frequency of 1 Hz and DC bias of 2 V. **c** *A*_R_ vs. time graph of samples 2.5 wt.% PVA-Mx sensor at different temperature exposure (20 °C, 40 °C, 60 °C, and 80 °C), under 10 kPa pressure at a frequency of 1 Hz and DC bias of 2 V

The reproducibility and mechanical stability are significant indicators of piezoresistive sensors performance. Hence, a test was performed to detect the responses of the 2.5 wt% PVA-Mx sensor capsule under continuous loading and unloading of 10 kPa pressure at a frequency of 1 Hz for 500 cycles and constant bias of 2 V. Figure 6a demonstrates that the fabricated sensor apprehended a consistent change in resistance during loading and unloading of 10 kPa pressure for 500 cycles. The inset of Fig. 6a shows the sensor response for the initial and final 30 cycles during the test. Compared to the initial 30 cycles, a slight drift in the resistance is observed for the last 30 cycles of pressure loading and unloading. However, a negligible change in relative resistance was noticed over the period of 500 cycles, which indicated the high reproducibility and robustness of the sensor capsule. Furthermore, most of the sensor response is significantly affected by the humidity and temperature of the surrounding environment. Thus, for the real-time working of the sensor, its stability towards the humidity and temperature of the surrounding environment plays a key role. We have analyzed the response of 2.5 wt% PVA-Mx sensor capsule in different humidity conditions at room temperature, under loading and unloading of 10 kPa pressure at a frequency of 1 Hz and DC bias of 2 V. Saturated salt solutions of calcium chloride, potassium carbonate, sodium chloride and distilled water were used to generate relative humidity (RH) environment of 16%, 43%, 75%, and 98%, respectively (Liu et al. 2019). The sensor response was monitored and change in relative resistance with respect to change in RH was analyzed, as depicted in Fig. 6b. Almost negligible change in relative resistance against RH was observed (Fig. 6b), and the sensor exhibited high stability towards change in RH. Furthermore, the sensor stability towards temperature was examined by exposing the sensor capsule to different temperature conditions. Figure 6c shows the change in relative resistance of the sensor with respect to change in temperature in the range of 20–80 °C. The sensor exhibited excellent stability towards the temperature variation. The sensor response repeatability under different temperature conditions is attributed to the high thermal stability of the silicon capsule that insulates the encapsulated PVA-Mx gel. The results demonstrated that change in RH and temperature of the surrounding medium has no considerable effect on the sensor response, which is desirable for practical applications.

**Fig. 7:**
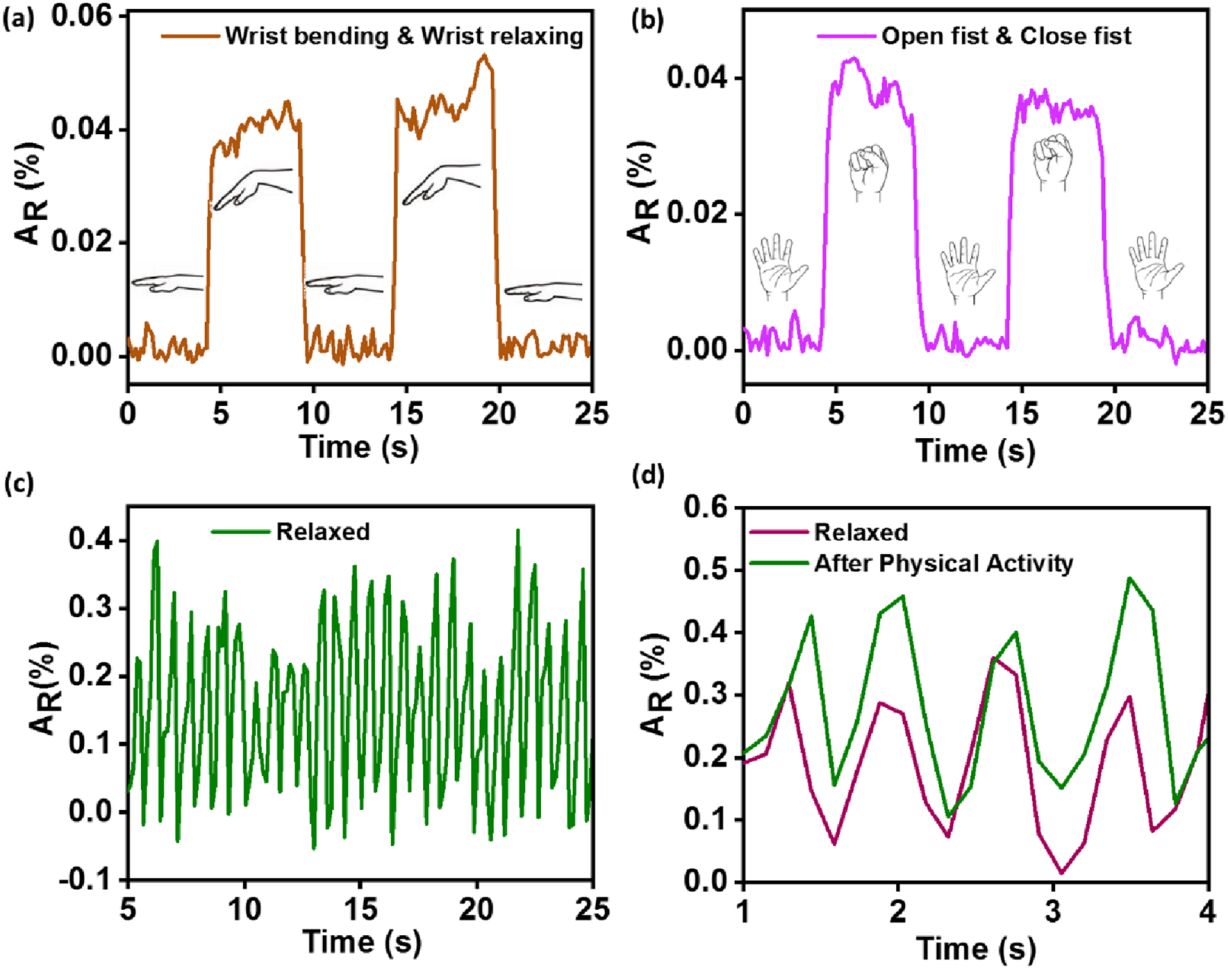
Applications of fabricated 2.5 wt% PVA-Mx composite-based piezoresistive sensor capsule in real-time monitoring of human activity and detection of **s**mall physical signals, evaluated under DC bias of 2 V. **a** Wrist bending. **b** Fist open and close. **c** Wrist pulse monitoring under relaxed conditions. **d** Wrist pulse of the subject under normal condition and after jogging measured after resting time of 5 min

To examine the application of fabricated sensor capsule, real-time monitoring of various human motions and pulse rates was performed. The sensor capsule (2.5 wt% PVA-Mx) was placed on a wrist joint using a masking adhesive tape for the signal response test. With a minor deflection in the human wrist, a change in resistance was observed, and a corresponding relative change with respect to the wrist bending cycle is shown in Fig. 7a. The wrist bending produces pressure on the sensor capsule, leading to a decrease in the resistance, which is depicted as a sudden surge in the relative resistance monitored during the wrist bending state. Furthermore, the response reached the initial state when the wrist was brought back to the relaxed position, as shown in Fig. 7a. Moreover, the sensor response was consistent for consecutive bending cycles. Furthermore, the sensor-generated similar response curve under the human motion of closing and opening the palm (Fig. 7b). To detect even weaker changes in pressure, the sensor was used to detect a physiological signal of the wrist pulse, as shown in Fig. 7c. The sensor apprehended symmetric trends of sharp peaks and measured 70 bpm (beats per minute). In general, the pulse rate ranges from 60 to 85 bpm in rest and varies with different physical activities. In another test, the wrist pulse was recorded in the rest position and 5 min after jogging, as shown in Fig. 7d. As the person under test was healthy, fast recovery of the pulse rate was noticed, and almost no change in heart rate was observed. However, compared to the pulse amplitude recorded at rest, a significant increase in the amplitude was displayed after jogging.

All the above characterization and analysis indicate that the 2.5 wt% PVA-Mx sensor capsule responded repeatedly and rapidly in real-time monitoring of the physiological status of the human body. The study reveals that the sensor has outstanding performance in the low-pressure regime, which implies that the fabricated sensor may find potential in wearable devices and could facilitate multi-functional detecting capabilities without compromising the comfort properties.

## Conclusions

In this study, a highly sensitive piezoresistive pressure sensor capsule was fabricated by PVA/MXene composite gel. The 2.5 wt% PVA/MXene sensor has a high sensitivity of 0.45 kPa^−1^ in a pressure range of 2–10 kPa and a fast response time of ~ 500 ms. The sensor capsule demonstrated excellent stability, reproducibility, and durability (over 500 loading and unloading cycles). The silicone capsule offered resistance to humidity and boosted the mechanical stability of the sensor. The present sensor can detect subtle human movement and facilitates real-time signal monitoring from wrist pulse. With ease in fabrication, high sensitivity, and stability, the reported piezoresistive sensor capsule is a powerful candidate for wearable electronics and medical monitoring.

## References

[CR1] Gao Y, Yan C, Huang H, Yang T, Tian G, Xiong D (2020). Microchannel-confined MXene based flexible piezoresistive multifunctional micro-force sensor. Adv Funct Mater.

[CR2] Jiang Q, Wu C, Wang Z, Wang AC, He JH, Wang ZL (2018). MXene electrochemical microsupercapacitor integrated with triboelectric nanogenerator as a wearable self-charging power unit. Nano Energy.

[CR3] Kharazmi A, Faraji N, Hussin R, Saion E, Mat Yunus WM, Behzad K (2015). Structural, optical, opto-thermal and thermal properties of ZnS–PVA nanofluids synthesized through a radiolytic approach. Beilstein J Nanotechnol.

[CR4] Lewis JA, Ahn BY (2015). Three-dimensional printed electronics. Nature.

[CR5] Li P, Zhao L, Jiang Z, Yu M, Li Z, Zhou X (2019). A wearable and sensitive graphene-cotton based pressure sensor for human physiological signals monitoring. Sci Rep.

[CR6] Li J, Fang L, Sun B, Li X, Kang SH (2020). Review—Recent progress in flexible and stretchable piezoresistive sensors and their applications. J Electrochem Soc.

[CR7] Liu H, Xiang H, Wang Y, Li Z, Qian L, Li P (2019). A flexible multimodal sensor that detects strain, humidity, temperature, and pressure with carbon black and reduced graphene oxide hierarchical composite on paper. ACS Appl Mater Interfaces.

[CR8] Ma Y, Liu N, Li L, Hu X, Zou Z, Wang J (2017). A highly flexible and sensitive piezoresistive sensor based on MXene with greatly changed interlayer distances. Nat Commun.

[CR9] Nan N, DeVallance DB (2017). Development of poly(vinyl alcohol)/wood-derived biochar composites for use in pressure sensor applications. J Mater Sci.

[CR10] Pan Y, Fu L, Zhou Q, Wen Z, Lin CT, Yu J (2020). Flammability, thermal stability and mechanical properties of polyvinyl alcohol nanocomposites reinforced with delaminated Ti_3_C_2_T_x_ (MXene). Polym Compos.

[CR11] Pang C, Lee GY, Kim T-i, Kim SM, Kim HN, Ahn SH (2012). A flexible and highly sensitive strain-gauge sensor using reversible interlocking of nanofibres. Nat Mater.

[CR12] Pei Y, Zhang X, Hui Z, Zhou J, Huang X, Sun G (2021). Ti_3_C_2_T_X_ MXene for sensing applications: recent progress, design principles, and future perspectives. ACS Nano.

[CR13] Qadir A, Le TK, Malik M, Amedome M, Dian KA, Saeed I, Yu Y (2021). Representative 2D-material-based nanocomposites and their emerging applications: a review. RSC Adv.

[CR14] Tang H, Zhuang S, Bao Z, Lao C, Mei Y (2016). Two-step oxidation of mxene in the synthesis of layer-stacked anatase titania with enhanced lithium-storage performance. ChemElectroChem.

[CR15] Wang K, Lou Z, Wang L, Zhao L, Zhao S, Wang D (2019). Bioinspired Interlocked structure-induced high deformability for two-dimensional titanium carbide (MXene)/natural microcapsule-based flexible pressure sensors. ACS Nano.

[CR16] Wang D, Zhang D, Li P, Yang Z, Mi Q, Yu L (2021). Electrospinning of flexible poly(vinyl alcohol)/MXene nanofiber-based humidity sensor self-powered by monolayer molybdenum diselenide piezoelectric nanogenerator. Nano-Micro Lett.

[CR17] Zhao L, Wang L, Zheng Y, Zhao S, Wei W, Zhang D (2021). Highly-stable polymer-crosslinked 2D MXene-based flexible biocompatible electronic skins for in vivo biomonitoring. Nano Energy.

